# Behavioral and histological analyses of the mouse *Bassoon* p.P3882A mutation corresponding to the human *BSN* p.P3866A mutation

**DOI:** 10.3389/fnins.2024.1414145

**Published:** 2024-07-26

**Authors:** Daiki Tanaka, Hiroaki Yaguchi, Kaichi Yoshizaki, Akihiko Kudo, Fumiaki Mori, Taichi Nomura, Jing Pan, Yasuo Miki, Hidehisa Takahashi, Taichi Hara, Koichi Wakabayashi, Ichiro Yabe

**Affiliations:** ^1^Department of Neurology, Faculty of Medicine and Graduate School of Medicine, Hokkaido University, Sapporo, Japan; ^2^Department of Disease Model, Aichi Developmental Disability Center, Kasugai, Japan; ^3^Integrated Analysis of Bioresource and Health Care, Future Medical Sciences, Kobe University Graduate School of Medicine, Kobe, Japan; ^4^Department of Neuropathology, Hirosaki University Graduate School of Medicine, Hirosaki, Japan; ^5^Department of Molecular Biology, Yokohama City University Graduate School of Medicine, Yokohama, Japan; ^6^Laboratory of Food and Life Science, Faculty of Human Sciences, Waseda University, Tokyo, Japan

**Keywords:** *Bassoon*, model mouse, behavioral analysis, histological analysis, progressive supranuclear palsy-like syndrome

## Abstract

Tauopathy is known to be a major pathognomonic finding in important neurodegenerative diseases such as progressive supranuclear palsy (PSP) and corticobasal degeneration. However, the mechanism by which tauopathy is triggered remains to be elucidated. We previously identified the point mutation c.11596C > G, p.Pro3866Ala in the *Bassoon* gene (*BSN*) in a Japanese family with PSP-like syndrome. We showed that mutated *BSN* may have been involved in its own insolubilization and tau accumulation. Furthermore, *BSN* mutations have also been related to various neurological diseases. In order to further investigate the pathophysiology of *BSN* mutation in detail, it is essential to study it in mouse models. We generated a mouse model with the mouse *Bassoon* p.P3882A mutation, which corresponds to the human *BSN* p.P3866A mutation, knock-in (KI) and we performed systematic behavioral and histological analyses. Behavioral analyses revealed impaired working memory in a Y-maze test at 3 months of age and decreased locomotor activity in the home cage at 3 and 12 months of age in KI mice compared to those in wild-type mice. Although no obvious structural abnormalities were observed at 3 months of age, immunohistochemical studies showed elevation of Bsn immunoreactivity in the hippocampus and neuronal loss without tau accumulation in the substantia nigra at 12 months of age in KI mice. Although our mice model did not show progressive cognitive dysfunction and locomotor disorder like PSP-like syndrome, dopaminergic neuronal loss was observed in the substantia nigra in 12-month-old KI mice. It is possible that BSN mutation may result in dopaminergic neuronal loss without locomotor symptoms due to the early disease stage. Thus, further clinical course can induce cognitive dysfunction and locomotor symptoms.

## Introduction

Progressive supranuclear palsy (PSP) is a clinical syndrome including supranuclear palsy, postural instability, and cognitive decline. Neuropathologically, PSP is defined by neuronal loss in the basal ganglia and brainstem with widespread occurrence of neurofibrillary tangles (NFTs; Williams and Lees, 2009; Yoshida et al., 2022) and accumulation of phosphorylated tau (p-tau) protein in the brain (Williams and Lees, 2009). For patients in whom the diagnosis is unclear, clinicians must continue to accurately describe the clinical situation in each individual instead of labeling them with inaccurate diagnostic categories such as atypical parkinsonism or PSP mimics (Williams and Lees, 2009). We previously identified the point mutation c.11596C > G, p.Pro3866Ala in the *Bassoon* gene (*BSN*) in a Japanese family with PSP-like syndrome (Yabe et al., 2018). We are the first in the world to report the involvement of BSN proteins in neurological diseases (Yabe et al., 2018). We showed that mutated BSN protein may be involved in its own insolubilization and tau accumulation (Yabe et al., 2018).

BSN is an active zone scaffolding protein and it has been suggested that BSN controls presynaptic autophagy (Okerlund et al., 2017; Hoffmann-Conaway and Brockmann, 2020; Montenegro-Venegas et al., 2020). It was also reported that there are associations between BSN and protein quality control systems including autophagy (Okerlund et al., 2017) and ubiquitination (Ivanova et al., 2016). Montenegro-Venegas et al. reported that BSN interacts directly with proteasome and inhibits proteasome activity via interaction with PSMB4 to control its activity at presynapses (Montenegro-Venegas et al., 2021). Moreover, Martinez et al. reported that *BSN* p.P3866A mutation caused tau seeding and showed toxicity in both mouse and Drosophila models for tauopathy and that BSN downregulation decreased tau spreading and overall disease pathology, rescuing synaptic and behavioral impairments and reducing brain atrophy (Martinez et al., 2022). It was shown that the degenerative eye phenotype was intensified in hTau-P301L flies when the BSN p.P3866A mutant was overexpressed (Martinez et al., 2022). Both WT BSN and mutant BSN p.P3866A interacted with tau in a Drosophila model (Martinez et al., 2022). Also, BSN overexpression in hTau-P301L flies led to an increase in tau-seeding activity, which was even higher with mutant *BSN* p.P3866A (Martinez et al., 2022). These studies suggested that BSN protein may play an important role in tauopathy.

The BSN protein and *BSN* gene were reported to be associated with multiple system atrophy (Hashida et al., 1998), Parkinson’s disease (PD; Andrews and Kukkle, 2023), Huntington’s disease (Huang et al., 2020), schizophrenia, bipolar disorder (Chen and Huang, 2021), multiple sclerosis (Schattling and Engler, 2019) and epilepsy (Ye et al., 2023). Gene burden analyses of rare, predicted deleterious variants provided evidence of *BSN* being linked to PD (Andrews and Kukkle, 2023). These studies have provided a mechanistic explanation for the recently described link between BSN and human diseases associated with pathological protein aggregation. That is why BSN may be one of the key proteins controlling neurological diseases.

To observe physiological changes caused by *BSN* mutations, we generated a mouse model of PSP-like syndrome with the mouse *Bassoon* p.P3882A mutation, which corresponds to the human Bassoon p.P3866A mutation.

## Materials and methods

### Animals

C57BL6/J male and female mice and ICR female mice were purchased from SLC (Hamamatsu, Japan). We used ICR mice for generating *Bassoon* p.P3882A mice. C57BL6/J male and female mice were used for mating. All experiments and analyses were performed with male C57BL6/J mice. For the generation of model mice with genome editing, 12-week-old males and 4-week-old females were used as sperm and oocyte donors, respectively. ICR female mice were used as recipients for embryo transfer. Male mice at 3 and 12 months of age were used for behavioral analyses. All animals were housed under a 12-h dark–light cycle (light from 7:00 to 19:00) at 23 ± 1°C with *ad libitum* access to food and water. All animal experiments were approved by the Ethics Committee for Animal Experiments of Aichi Institute for Developmental Research (2020-005) and Hokkaido University Graduate School of Medicine (22-0042) and were carried out in accordance with the National Institutes of Health guide for the care and use of laboratory animals.

### Generation of *Bassoon* p.P3882A mice

Briefly, pronuclear stage embryos were produced using *in vitro* fertilization. Alt-R™S.p. Cas9 nuclease V3 (Cat#1081058), crRNA, and Alt-R®CRISPR-Cas9 tracrRNA (cat#1072532) were obtained from Integrated DNA Technologies Inc. (Coralville, IA, USA). crRNA was designed to target the *Bassoon* gene of C57BL6/J mice (5′-CCAGAGTACTCAGAGCAATCTCT-3′). Single-stranded oligodeoxyribonucleotides (ssODNs) consisting of 70-bps homology arms flanking the mutation c.11596C > G, p.Pro3866Ala and a modified restricted enzyme site for the *Sca* I site were synthetized by IDT (Coralville, IA, USA). The sequence was as follows: p.Pro3866Ala (Bold)/modified restricted enzyme site for the *Sca* I site (underline), 5′-CAAAGCGCCCCAGCAGGGACGGGCTCCTCAG GCGCAGACAACTCCAGGAGCTGGACCTGCAGGTGAGCTGTGCCCAGAGATCTCAGAGCAATCTCTCCCTATACCACTGT-3′. Nucleases were introduced into pronuclear stage embryos using the modified TAKE method (Kaneko and Mashimo, 2015). Two-cell embryos were transferred into the oviducts of pseudo-pregnant ICR females that were mated with vasectomized males the day before embryo transfer.

### Behavioral analyses

Systematic behavioral analyses were performed according to previous reports with a total of 44 mice (WT mice at 3 months of age: 11, WT mice at 12 months: 11, KI mice at 3 months: 11, KI mice at 12 months: 11; Yoshizaki et al., 2008; Takagi et al., 2015; Yoshizaki and Asai, 2020; Yoshizaki and Kimura, 2021). Because two of the KI mice at 12 months died due to a water supply problem, we used 42 mice for analysis.

### Open-field test

Locomotor activity and anxiety-like behavior were assessed by using a square open-field apparatus (50 × 50 × 50 cm, O’Hara & Co., Ltd., Tokyo, Japan) according to our previous report (Yoshizaki and Asai, 2020). Each mouse was placed in the center of the apparatus. The center zone was defined as a square 10 cm area away from the wall. The distance traveled and the time spent in the center zone were recorded for 10 min with a video imaging system (EthoVisionXT; Noldus Information Technology), according to previous papers.

### Rotarod test

Motor coordination and learning were assessed by a rotarod test (single-lane apparatus, MK-630B; Muromachi, Tokyo, Japan). Each mouse was placed on a rotarod with a progressive acceleration setting from 4 to 40 rpm over a 10-min period. Five trials were performed for two consecutive days (3 trials on Day 1 and 2 trials on Day 2 at intervals of at least 1 h), and the time until falling was recorded.

### Home cage activity test

Home cage activity was measured by the rotational frequency of a running wheel. Each mouse was placed alone in a home cage (180 mm in width × 225 mm in depth × 123 mm in height) with a running wheel (50 mm in width × 143 mm) under a 12-h light–dark cycle (lights on at 07:00 h) and had free access to both food and water. The rotational frequency of the running wheel was automatically recorded with a porcelain sensor (RWC-15; Melquest Ltd., Toyama, Japan) for 5 consecutive days starting at 13:00 on each day (CIF-4; Actmaster, Melquest, Ltd., Toyama, Japan).

### Y-maze test

Spatial working memory and exploratory activity were assessed by using a Y-maze apparatus (arm length: 40 cm, arm bottom width: 3 cm, arm upper width: 13 cm, height of wall: 15 cm, BrainSicence Idea, Osaka, Japan). Each mouse was placed in the center area. The number of entries into arms and alterations were recorded for 10 min with a video imaging system (EthoVisionXT; Noldus information Technology). Working memory was calculated as: “number of correct alterations” divided by “number of total arm entries.”

### Novel object recognition test

Non-spatial working memory was assessed by a novel object recognition test in an open field apparatus (50 × 50 × 50 cm, O’Hara & Co., Ltd., Tokyo, Japan). The objects were made of urethane or metal and had two different shapes and colors: sphere (white) and cylinder (silver). In the first trial on the first day, two identical objects were presented on opposite sides of the apparatus, and the mice were allowed to explore the objects for 10 min. Exploration was considered as directing the nose at a distance of 1 cm from the object. In the second trial on the next day, one of the objects presented in the first trial was replaced with a novel object and the mice were placed in the box for 3 min. The time spent exploring the familiar (F) object and the time spent exploring the novel (N) object were automatically recorded with a video imaging system (EthoVisionXT; Noldus information Technology). A discrimination index was calculated as (N − F)/(N + F). Care was taken to avoid place preference and olfactory stimuli by randomly changing the role (which object is familiar or novel) and positions of the two objects during the second trial and by cleaning them carefully with 70% alcohol.

### Histopathological analysis

For immunohistochemical analysis, mice were deeply anesthetized with isoflurane and perfused with 4% paraformaldehyde (PFA) dissolved in phosphate-buffered saline (PBS). Brains were postfixed with the same fixative overnight and then washed with PBS. Histopathological analysis was carried out as reported previously (Tanaka et al., 2022). The cerebrum was anteriorly transected at 1 mm anterior to the anterior edge of the pons and sliced at 2-mm intervals anterior and posterior to this level. The cerebellum and brainstem were sectioned in a midsagittal section. After dehydration through a graded ethanol series, the samples were embedded in paraffin and cut into 4-μm-thick sections. For routine histological examination, sections from each case were stained with hematoxylin and eosin (HE).

### Immunohistochemistry

Immunohistochemical analysis was carried out using the above paraffin-embedded sections. The sections were dehydrated and pretreated with heat retrieval using an autoclave for 15 min in 10 mM citrate buffer (pH 6.0) for primary antibodies. The sections were then subjected to immunohistochemical processing using the avidin–biotin-peroxidase complex (ABC) method with a Vectastain ABC kit (Vector, Burlingame, CA) and diaminobenzidine (DAB; Sigma, St. Louis, MO). DAB exposure time was the same for each slide glass. In addition, the sections were counterstained with hematoxylin. We used primary antibodies against BSN (mouse, ab82958, Abcam, Cambridge, UK; 1:100), p-tau (rabbit, ab151559, Abcam; 1:2,000), synaptophysin (mouse, SY38, Dako, Glostrup, Denmark; 1:1,000), alpha-synuclein (mouse, 2A7, Novus Biologicals, Centennial, CO; 1:1,000), tyrosine hydroxylase (TH; mouse, TH-16, Sigma, St. Louis, MO; 1:500), TH (rabbit, CA-101, Protos Biotech Corporation, New York, NY; 1:200), ubiquitin (rabbit, Z0458, Dako; 1:1,000), p62 (mouse, 610,832, BD Biosciences, Franklin Lakes, NJ; 1:100), and vesicular monoamine transporter 2 (VMAT2) antibody (rabbit, 20,873-1-AP, Proteintech, Rosemont, IL, USA; 1:100). All regions of wild-type (WT) and *Bsn* knock-in (KI) mice were examined histopathologically, since BSN immunoreactivity was increased in the hippocampus of KI mice at the age of 12 months.

### Semi-quantitative analysis of immunoreactivity for the proteins

Digital images of the caudate-putamen were captured by a digital camera (Provis AX-70, Olympus, Tokyo, Japan) and evaluated semi-quantitatively in terms of gray levels with NIH Image (version 1.61). The data were normalized by subtracting the background. The values were analyzed using Student’s *t*-test to examine differences in immunoreactivity for the proteins (BSN, p-tau, TH, synaptophysin, and alpha-synuclein) in the caudate-putamen between WT and KI mice.

### Cell counts of TH-positive neurons

In each mouse, the numbers of TH-positive neurons were counted on both sides of the substantia nigra immunostained with TH-16. This primary antibody works well for both human and mouse samples. The entire substantia nigra was surveyed at a magnification of 200× using an eyepiece graticule and parallel sweeps of the microscope stage. The numbers were analyzed using Student’s *t*-test to examine the differences between WT and KI mice.

### Statistical analysis

Data are presented as means ± standard error of the mean (SEM). The differences between results of behavioral analyses for wild-type and *Bsn* variant mice were determined by Student’s *t*-test and two-way ANOVA. StatPlus was used for statistical analysis and a *p* value less than 0.05 was considered to be statistically significant. Mann–Whitney’s U test was used for statistical comparison of immunostaining intensities.

## Results

### Generation of the mouse *Bsn* p.P3882A variant that corresponds to the human *BSN* p.P3866A

In order to examine the effect of human *BSN* p.P3866A on the pathogenesis of PSP-like syndrome, we generated a mouse *Bsn* p.P3882A variant (corresponding to human *BSN* p.P3866A, Supplemental Figure 1A) knock-in mouse by using the TAKE (Technique for Animal Knockout system by Electroporation) method as previously described. Briefly, we designed gRNA targeting the coding sequence of *Bsn* and ssODNs with *Bsn* p.P3882A mutation and modified restricted enzyme site for *Sca* I (Supplemental Figure 1B). *Bsn* variant genotyping was conducted using PCR and restriction enzyme with *Sca* I. WT alleles were digested with *Sca* I, while *Bsn* variant alleles were not (Supplemental Figure 1C).

### Impaired locomotion and impaired spatial working memory in 3-month-old *Bsn* variant mice

**Figure 1:**
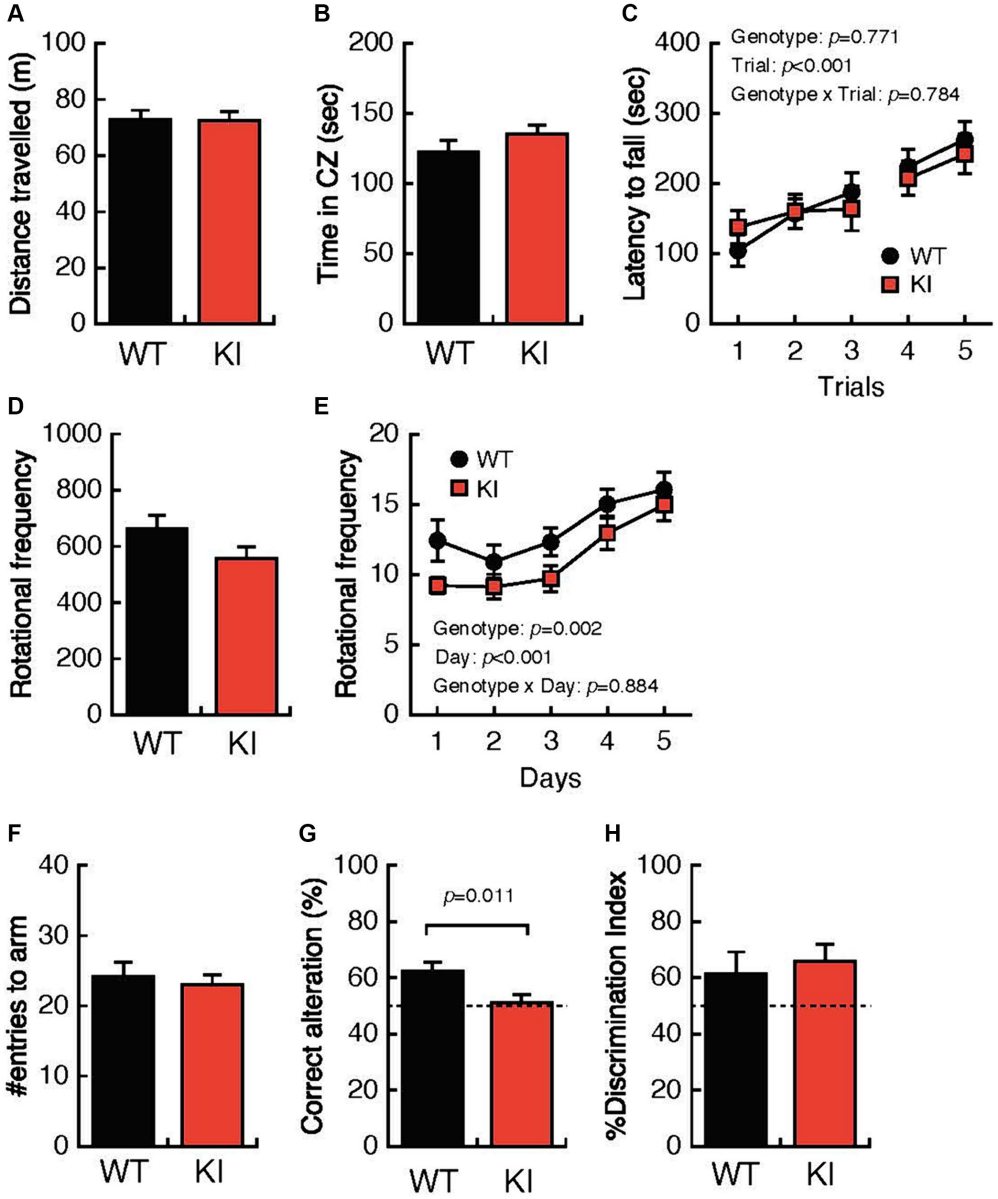
Impaired locomotion and spatial working memory in 3-month-old Bsn variant mice. **(A,B)** Both the distance traveled and the time spent in the center zone in the open-field test were comparable in 3-month-old WT mice and 3-month-old KI mice. **(C)** The mean latency until falling from the rotating rod was not different in WT mice and KI mice. **(D,E)** Daily rotational frequency, but not total rotational frequency, in the home cage activity test was significantly decreased in KI mice compared with that in WT mice. **(F)** The total number of entries to the arm in the Y-maze test was not different in KI mice. **(G)** Percentages of correct alterations in the Y-maze test were significantly decreased in KI mice compared with those in WT mice. **(H)** Abilities for discrimination of the familiar object and the novel object were comparable in WT mice and KI mice. WT, wild-type mice; m/m, homozygous KI mice; CZ, center zone.

Both the distance traveled and the time spent in the center zone in the OFT were comparable for 3-month-old WT mice and 3-month-old KI mice (Figures 1A,B). Likewise, the mean latency until falling from the rotating rod in the RRT was not different for WT mice and KI mice (Figure 1C). In contrast, daily rotational frequency in the home cage was significantly decreased for KI mice compared with that for WT mice (Figures 1D,E). Intriguingly, the ratio of correct alterations in the YMZ test was significantly decreased for KI mice compared with that for WT mice (Figure 1G), although the total number of entries to the arm in the YMZ test was not different for KI mice (Figure 1F). In contrast, abilities for discrimination of the familiar object and novel object in the NORT were comparable for WT mice and KI mice (Figure 1H). These results suggest decreased locomotor activity in the acclimated home cage and impaired spatial working memory in young KI mice.

### Pathological analyses of the *Bsn* p.P3882A mutation in 3-month-old mice

**Figure 2:**
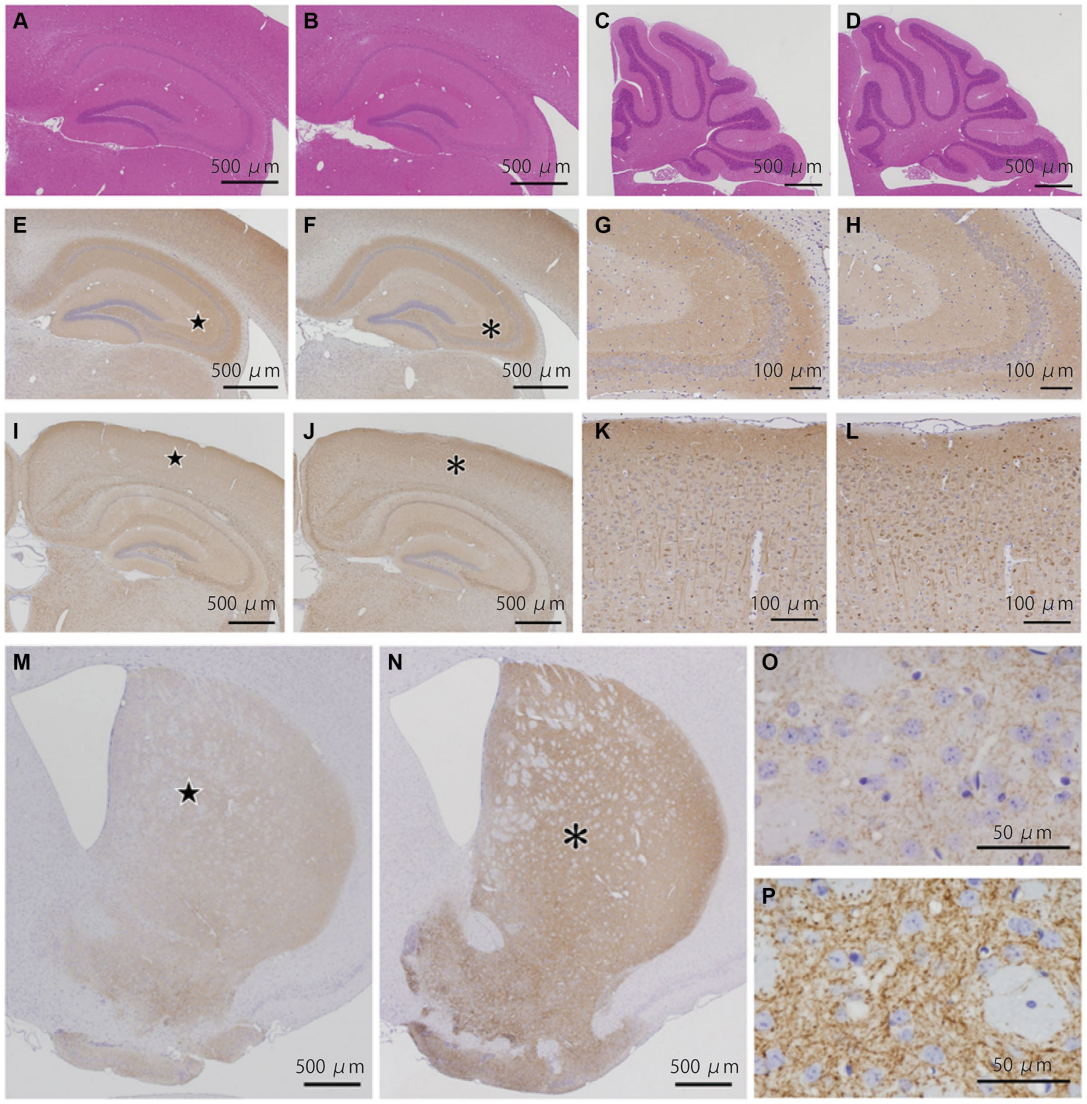
Histopathological analyses of the *Bsn* p.P3882A mutation in 3-month-old mice. **(A–D)** HE staining in the hippocampus **(A,B)** and cerebellum **(C,D)** of WT mice **(A,C)** and KI mice **(B,D)**. No apparent abnormality in WT and KI mice. **(E–H)** Immunoreactivity for bassoon in the hippocampus of WT **(E,G)** and KI mice **(F,H)**. Higher-magnification view of the area indicated by the star in **(E)** and asterisk in **(f)** showing similar fine granular staining in the CA2-3 region of WT **(G)** and KI mice **(H)**. **(I–L)** Immunoreactivity for p-tau in the hippocampus of WT **(I,K)** and KI mice **(J,L)**. Higher-magnification view of the area indicated by the star in **(I)** and asterisk in **(J)** showing similar staining patterns in the neocortex of WT **(K)** and KI mice **(L)**. **(M–P)** Immunoreactivity for TH (TH-16) in the striatum of WT **(M,O)** and KI mice **(N,P)**. TH immunoreactivity in the striatum of KI mice was much stronger than that in the striatum of WT mice. Higher-magnification view of the area **(P)** indicated by the asterisk in **(N)** of KI mice showing much stronger immunoreactivity of nerve cell processes than that of the area **(O)** indicated by the star in **(M)** of WT mice. WT, wild-type mice at 3 months of age; KI, *Bsn* knock-in mice at 3 months of age.

We examined 5 WT and 4 KI mice (Supplemental Table 1). HE staining of the cerebrum, cerebellum, and brainstem showed no structural abnormalities or neuronal loss in WT and KI mice (Figures 2A–D; Supplemental Figures 2A,B). Immunostaining for BSN (Figures 2E–H), p-tau (Figures 2I–L), synaptophysin (Supplemental Figures 2C,D), and alpha-synuclein (Supplemental Figures 2E,F) showed no obvious differences between WT and KI mice. No abnormal structures were found in sections stained with antibodies against p-tau (Figures 2I–L), ubiquitin, or p62. Immunostaining with a TH monoclonal antibody (TH-16) showed that TH immunoreactivity in the striatum of KI mice was stronger than that in the striatum of WT mice (Figures 2M–P), and significant differences were observed by Student’s t test (*p* < 0.01; Supplemental Table 1; Supplemental Figure 3C). A similar trend was observed in immunostaining with a TH polyclonal antibody (CA-101; Supplemental Figure 4), but the difference was not statistically significant. There was no significant difference in the number of TH-positive neurons in the substantia nigra between KI and WT mice at 3 months (Supplemental Table 1; Supplemental Figures 3F, 5). The immunoreactivity of VMAT2 in the striatum of KI mice was weaker than that of WT mice at the age of 3 months (Supplemental Figures 9A–D).

### Impaired locomotion in 12-month-old *Bsn* variant mice

**Figure 3:**
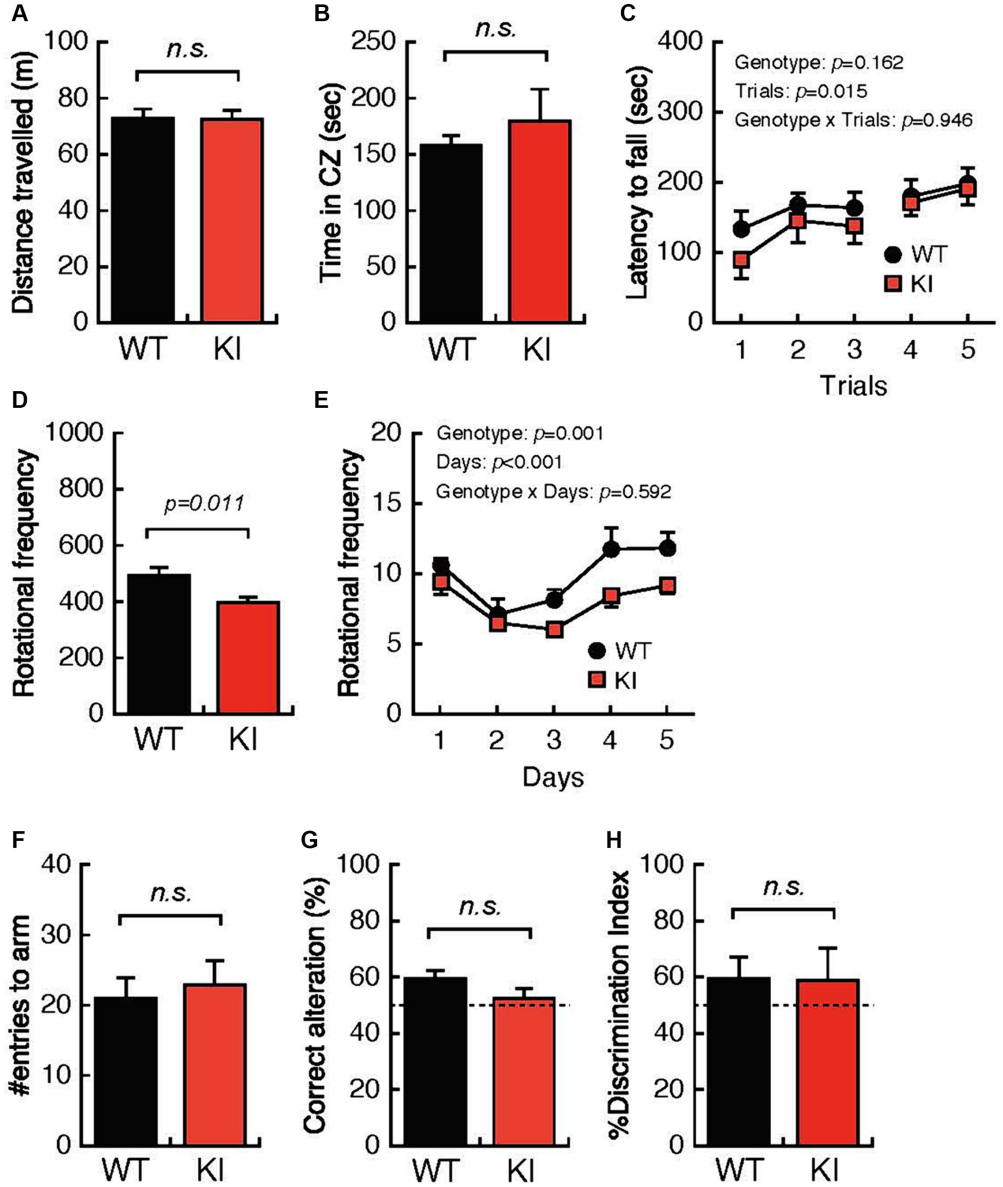
Impaired locomotion and spatial working memory in 12-month-old *Bsn* variant mice. **(A,B)** Both the distance traveled and the time spent in the center zone in the open-field test were comparable in 12-month-old WT mice and 12-month-old KI mice. **(C)** The mean latency until falling from the rotating rod was not different in WT mice and KI mice. **(D,E)** Daily rotational frequency, but not total rotational frequency, in the home cage activity test was significantly decreased in KI mice compared with that in WT mice. **(F,G)** Percentages of correct alterations in the Y-maze test were not significantly decreased in KI mice compared with those in WT mice. **(H)** Abilities for discrimination of the familiar object and the novel object were comparable in WT mice and KI mice. WT, wild-type mice; m/m, homozygous *Bsn* KI mice.

We also performed systematic behavioral analyses using identical WT mice and KI mice at 12 months of age. WT mice and KI mice showed comparable total distances traveled and times spent in the center zone in the OFT (Figures 3A,B) and comparable mean latencies until falling from the rotating rod in the RRT (Figures 3C). In contrast, daily rotational frequency in the home cage was significantly decreased for KI mice compared with that for WT mice (Figures 3D,E). Working memory and cognitive function in the YMZ test (Figures 3F,G) and the NORT (Figure 3H) were not different in WT mice and KI mice. These results suggest decreased locomotor activity in the acclimated home cage in aged KI mice.

### Pathological analyses of the *Bsn* p.P3882A mutation in 12-month-old mice

**Figure 4:**
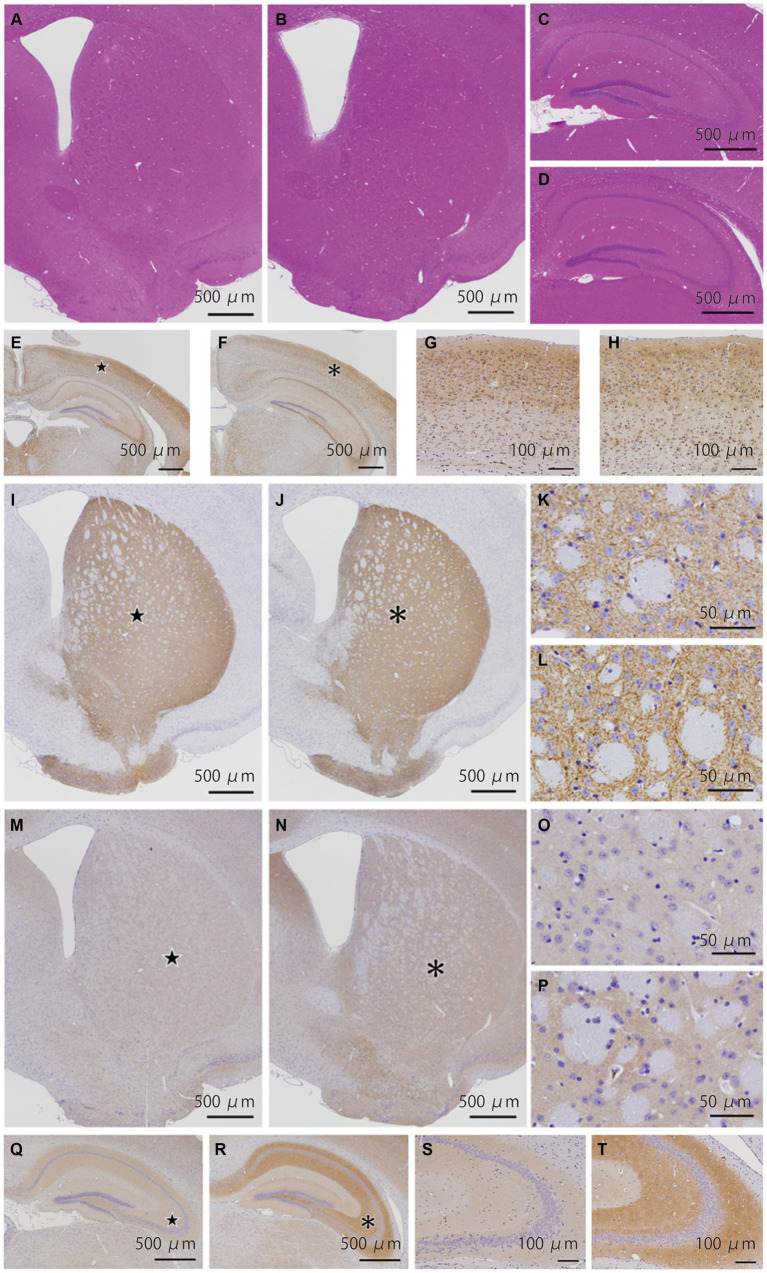
Histopathological analyses of the *Bsn* p.P3882A mutation in 12-month-old mice. **(A–D)** HE staining in the striatum **(A,B)** and hippocampus **(C,D)** of WT **(A,C)** and KI mice **(B,D)**. No apparent abnormality in WT and KI mice. **(E–H)** Immunoreactivity for p-tau in the hippocampus of WT **(E,G)** and KI mice **(F,H)**. Higher-magnification view of the area indicated by the star in **(E)** and asterisk in **(F)** showing similar staining patterns in the neocortex of WT **(G)** and KI mice **(H)**. **(I–L)** Immunoreactivity for TH (TH-16) in the striatum of WT **(I,K)** and KI mice **(J,L)**. Higher-magnification view of the area indicated by the star in **(I)** and asterisk in **(J)** showing similarly strong immunoreactivity of nerve cell processes. **(M–P)** Immunoreactivity for bassoon in the striatum of WT **(M,O)** and KI mice **(N,P)**. **(O,P)** Higher-magnification view of the area indicated by the star in **(O)** and asterisk in **(P)**. Immunoreactivity of the gray matter in KI mice was slightly stronger than that in WT mice. **(Q–T)** Immunoreactivity for bassoon in the hippocampus of WT **(Q,S)** and KI mice **(R,T)**. **(S,T)** Higher-magnification view of the area indicated by the star in **(Q)** and asterisk in **(R)**. Immunoreactivity of the molecular and polymorphic layers in KI mice was stronger than that in WT mice. WT, wild-type mice at 12 months of age; KI, *Bsn* knock-in mice at 12 months of age.

We examined 8 WT and 6 KI mice (Supplemental Table 2). HE staining of the striatum (Figures 4A,B), hippocampus (Figures 4C,D), cerebellum (Supplemental Figures 6A,B), and brainstem (Supplemental Figures 6C,DS1) showed no structural abnormalities in WT and KI mice. Immunostaining for p-tau (Figures 4E–H), synaptophysin, alpha-synuclein (Supplemental Figure 7), and TH (Figures 4I–L), showed no obvious differences between WT and KI mice in the cerebrum, cerebellum and brainstem. No abnormal structures such as neuronal or glial inclusions were observed (Supplemental Figure 8). BSN immunohistochemistry showed stronger staining in the cerebral cortex, striatum (Figures 4M–P), and hippocampus (Figures 4Q–T) in KI mice than in WT mice. Semi-quantitative analysis in the striatum showed significant differences (*p* < 0.01) in Student’s *t*-test (Supplemental Table 2; Supplemental Figure 3A). Furthermore, the number of TH-positive neurons in the substantia nigra of KI mice was significantly decreased compared to that in the substantia nigra of WT mice at 12 months of age (*p* < 0.01; Supplemental Table 2; Supplemental Figures 3F, 5). The immunoreactivity of VMAT2 in the striatum of KI mice was weaker than that of WT mice at the age of 12 months (Supplemental Figure 9E–H).

## Discussion

There are three important findings in the present study. First, we generated mice with *Bsn* p.P3882A mutation that corresponds to the human *BSN* p.P3866A. Second, at 3 months and 12 months of age, the HCA test showed significant differences between WT mice and KI mice. Third, immunostaining using a BSN monoclonal antibody showed stronger staining in the cerebral cortex, striatum, hippocampus in KI mice than in WT mice at 12 months of age. TH-positive neuronal loss was observed in the substantia nigra in KI mice at 12 months of age. These findings may suggest changes in dopaminergic innervation and BSN expression.

We previously identified the point mutation c.11596C > G, p.Pro3866Ala in *BSN* in a Japanese family with PSP-like syndrome (Yabe et al., 2018). Domains of the *BSN* p.P3866A mutation were reported to be conserved evolutionarily (Yabe et al., 2018). Mutated BSN (p.Pro3866Ala) may be involved in its own insolubilization and tau accumulation (Yabe et al., 2018). Our report led to a series of reports linking *BSN* and neurological diseases. Using *BSN* knockout mice, it has been shown that *BSN* inhibits proteasome activity (Montenegro-Venegas et al., 2021). It has also been reported that the p.Pro3866Ala mutation exacerbates tau seeding in a Drosophila model (Martinez et al., 2022). These findings strongly indicate a notable link between the presynaptic active zone and neurodegenerative diseases. Alpha-synuclein and tau are important for the maintenance of synaptic vesicle function (Lv et al., 2022). Furthermore, those reports indicate that BSN may be a key molecule in protein accumulation. From the above, it is important to examine what pathological significance the *BSN* mutation, which we have already shown to be pathogenic in our immortalized cell lines, has in actual animal models. Significant differences in the results of behavioral analysis were observed at 3 months and 12 months of age and TH-positive neuronal loss was observed at 12 months of age in our mouse model, suggesting that this mutation plays an important role *in vivo*. Working memory was impaired in *Bsn* p.P3882A variant mice at 3 months of age but not at 12 months of age. The impairment of working memory in KI mice at 3 months of age may be due to enhanced immunoreactivity of TH. Some reports have suggested that excessive dopamine impairs spatial working memory (Zahrt et al., 1997; Vijayraghavan et al., 2007). Therefore, appropriate dopamine balance may be essential for maintenance of healthy working memory. In our KI mice, 3-month-old KI mice showed loss of spontaneous activity in the HCA test and early dopaminergic activation. These results indicate the possibility that 3-month-old KI mice showed cognitive impairment due to inappropriate dopamine regulation. Although decrease tendency was also observed in 12-month-old KI mice, the differences did not reach to significance. Since the TH-positive neurons significantly decrease in 12-month-old KI mice, it is possible that cognitive impairment may develop in further aged KI mice. Further study is needed.

Our KI mice showed TH-positive neuronal loss at 12-month-old, while elevation of TH immunoreactivity at 3-month-old. Also, decrease immunoreactivities of VMAT2 were observed in both 3 and 12 months old KI mice. In humans, decrease immunoreactivity of VMAT2 was followed by decrease immunoreactivity of TH (Kang et al., 2021). Moreover, it has been reported that decrease of VMAT2 results in neuronal loss in the substantia nigra in aged mice probably due to oxidative stress from increasing dopamine metabolites in cytosol (Caudle et al., 2007; Guillot and Miller, 2009), and therefore, VMAT2 plays an important role for neuroprotection in dopaminergic neurons. We hypothesized that *BSN* mutation may prevent dopamine packaging and releasing through dysfunction of VMAT2. TH immunoreactivity increased possibly due to inhibition of dopamine releasing in 3-month-old KI mice. Accumulating the stress of dysfunction of packaging and releasing of dopamine may result in increasing neuronal toxicity and neuronal loss in 12-month-old KI mice. Further studies for elucidating the relationship between mutant BSN and VMAT2 are needed.

Elevated BSN at the protein level at 12 months of age may suggest that BSN proteinopathy is associated with cognitive dysfunction. In this study, *Bsn* KI mice showed TH-positive neuronal loss without tauopathy at 12 months of age. Also, no accumulation of alpha-synuclein was observed. The patient with Parkinson disease, one of the most studied neurodegenerative diseases with dopaminergic neuronal loss, shows locomotor symptoms several to two decades of years after onset (Kalia and Lang, 2015). Due to difficulty in diagnosis as Parkinson disease without locomotor symptoms, the pathological changes in the early stage of Parkinson disease have been unknown. This is also the case with other neurodegenerative diseases. Since our previous *in vitro* study showed that mutant Bsn expression induces insolubilized tau accumulation (Yabe et al., 2018), it is possible that older KI mice can represent tauopathy. Other hypothesis is that the mechanism other than protein aggregation, can lead to neuronal loss. In fact, *BSN* mutation was also associated with various neurological diseases without protein aggregation, such as schizophrenia (Chen and Huang, 2021), multiple sclerosis (Schattling and Engler, 2019), and epilepsy (Ye et al., 2023). These reports suggest that *BSN* mutation may be related to the vulnerability of neurons in the central nervous system. Further electrophysiological and biochemical analyses and longer time-course observation are needed to elucidate the mechanism in the future.

In conclusion, the generation of *Bsn* KI mice in this study can be considered a first step toward future important research. Although no structural abnormalities were observed at 3 months and 12 months of age, the HCA test showed slightly significant differences between WT mice and KI mice. Elevated Bsn at the protein level at 12 months of age suggests that BSN proteinopathy may be a gain of function. Unlike our previous report of patients with PSP-like syndrome with *BSN* mutation, our mouse model did not show tauopathy at 12-month-old, but showed dopaminergic neuronal loss. Since *BSN* mutation has been closely related to various neurological diseases, our model can become a useful tool for studying the mechanism for neurodegeneration. Therefore, we will continue to conduct experiments to determine how BSN dysfunction causes neuronal loss. Further elucidation of the molecular and biological mechanisms may lead to the identification of target proteins for treatment of neurological diseases.

### Limitations

There are two main limitations of this study. First, protein expression including expression of tyrosine hydroxylase, BSN and p-tau was evaluated only by tissue staining and not by biochemical evaluation. Second, behavioral and histological analyses were carried out only up to the age of 12 months.

## Data availability statement

The raw data supporting the conclusions of this article will be made available by the authors, without undue reservation.

## Ethics statement

The animal studies were approved by the Ethics Committee for Animal Experiments of Aichi Institute for Developmental Research (2020-005) and Hokkaido University Graduate School of Medicine (22-0042). The studies were conducted in accordance with the local legislation and institutional requirements. Written informed consent was obtained from the owners for the participation of their animals in this study.

## Author contributions

DT: Data curation, Investigation, Writing – original draft, Writing – review & editing. HY: Data curation, Investigation, Writing – original draft, Writing – review & editing, Conceptualization, Methodology, Project administration. KY: Investigation, Writing – original draft. AK: Investigation, Methodology, Writing – review & editing. FM: Investigation, Methodology, Writing – review & editing. TN: Investigation, Writing – review & editing. JP: Investigation, Writing – review & editing. YM: Investigation, Writing – review & editing. HT: Supervision, Writing – review & editing. TH: Investigation, Methodology, Writing – review & editing. KW: Supervision, Writing – review & editing, Investigation. IY: Data curation, Formal analysis, Funding acquisition, Project administration, Supervision, Writing – review & editing.

## Supplementary material

SUPPLEMENTARY FIGURE S1 Generation of the mouse *Bsn* p.P3882A mutation that corresponds to the human BSN p.P3866A. **(a)** Evolutionarily conserved domains of the BSN p.P3866A mutation. **(b)** We generated the mouse *Bsn* p.P3882A mutation that corresponds to the human BSN p.P3866A. **(c)** Genotypes were determined by PCR using primers designed basically on the same site by Sca1. WT, wild-type mice; KI, *Bsn* knock in mice.

SUPPLEMENTARY FIGURE S2 Histopathological analyses of the *Bsn* p.P3882A mutation in 3-month-old mice. **(a,b)** HE staining in the brainstem of WT **(a)** and KI mice **(b)**. No apparent abnormality in WT and KI mice. **(c–f)** Immunoreactivity for alpha-synuclein in the cortex and the hippocampus of WT **(c)** and KI mice **(d)**. Immunoreactivity for synaptophysin in the cortex and the hippocampus of WT **(e)** and KI mice **(f)**. WT, wild-type mice at 3 months of age; KI, *Bsn* knock-in mice at 3 months of age. No abnormal structures were found in sections.

SUPPLEMENTARY FIGURE S3 Semi-quantitative analysis for immunoreactivity and nerve cell counts. Semi-quantitative analysis for immunoreactivity for bassoon (BSN, **a**), phosphorylated tau (p-Tau, **b**), tyrosine hydroxylase (TH-16, **c**), synaptophysin (SYP, **d**) and alpha-synuclein (SYN, **e**) in the caudate-putamen and cell counts of TH-positive neurons in the substantia nigra **(f)**. BSN immunoreactivity in KI mice was more intense than that in WT mice at 12 months of age (*p* <0.01) **(a)**. TH immunoreactivity in KI mice was more intense than that in WT mice at 3 months of age (*p* <0.01) **(c)**. The number of TH-positive neurons in the substantia nigra of KI mice was significantly decreased compared to that in the substantia nigra of WT mice at 12 months of age (*p* <0.05) **(f)**. OD, optical density.

SUPPLEMENTARY FIGURE S4 Immunoreactivity for TH (CA-101) in the striatum of 3-month-old mice. Immunoreactivity for TH (CA-101) in the striatum of WT **(a,c)** and KI mice **(b, d)**. TH immunoreactivity in the striatum of KI mice was slightly stronger than that in the striatum of WT mice. Higher-magnification view of the area **(d)** indicated by the asterisk in **(b)** of KI mice showing slightly stronger immunoreactivity of nerve cell processes than that of the area **(c)** indicated by the star in **(a)** of WT mice. WT, wild-type mice at 3 months of age; KI, *Bsn* knock-in mice at 3 months of age.

SUPPLEMENTARY FIGURE S5 TH immunohistochemistry (TH-16) in the substantia nigra. Immunohistochemistry for TH in the substantia nigra of WT **(a,c)** and KI mice **(b,d)** at 3 and 12 months of age. TH-immunoreactive neurons in the substantia nigra appear to be decreased in KI mice compared to that in WT mice at 12 months of age.

SUPPLEMENTARY FIGURE S6 Histopathological analyses of the *Bsn* p.P3882A mutation in 12-month-old mice. **(a,b)** HE staining in the cerebellum of WT **(a)** and KI mice **(b)**, and that in the brainstem of WT **(c)**, and KI mice **(d)**. No apparent abnormality in WT and KI mice.

SUPPLEMENTARY FIGURE S7 Alpha-synuclein immunoreactivity in the striatum of 3- and 12-month-old mice. There was no significant difference in alpha-synuclein immunoreactivity in the striatum between WT **(a,c)** and KI mice **(b,d)** at 3 months of age as well as between WT **(e,g)** and KI mice **(f,h)** at 12 months of age. Higher-magnification view of the area **(c,d,g,h)** indicated by the star in **(a,e)** and asterisk in **(b,f)**. Because immunostaining treatments of sections from 3- and 12-month-old mice were performed separately, differences between 3- and 12-month-old mice were not examined.

SUPPLEMENTARY FIGURE S8 Immunohistochemistry for BSN, p-Tau, ubiquitin and p62 in the hippocampus of KI mice at 12 months of age. Immunohistochemistry for BSN **(a,b)**, p-Tau **(c,d)**, ubiquitin **(e,f)** and p62 **(g,h)** in the hippocampus of KI mice at 12 months of age. Right panels **(b,d,f,h)** are higher magnification views of the regions indicated by asterisks in left panels **(a,c,e,g)**. No pathological lesions, such as neuronal or glial inclusions, were observed.

SUPPLEMENTARY FIGURE S9 Vesicular monoamine transporter 2 (VMAT2) immunoreactivity in the striatum of 3- and 12-month-old mice. Immunoreactivity for VMAT2 in the striatum of WT **(a,c)** and KI mice **(b,d)** in the 3-month-old. VMAT2 immunoreactivity in the striatum of KI mice was weaker than that in the striatum of WT mice. Higher-magnification view of the area in 3-month-old KI mice **(d)** showing weaker immunoreactivity of nerve cell processes than that of the area in 3-month-old WT mice **(c)**. Immunoreactivity for VMAT2 in the striatum of WT **(e,g)** and KI mice **(f,h)** in the 12-month-old. VMAT2 immunoreactivity in the striatum of KI mice was weaker than that in the striatum of WT mice. Higher-magnification view of the area in 12-month-old KI mice **(h)** showing weaker immunoreactivity of nerve cell processes than that of the area in 12-month-old WT mice **(g)**. WT, wild-type mice at 12 months of age; KI, *Bsn* knock-in mice at 12 months of age.
