# Supplementary material for: Behavioral and histological analyses of the mouse Bassoon p.P3882A mutation corresponding to the human BSN p.P3866A mutation
Source: Front Neurosci. 2024 Jul 26;18:1414145. doi: 10.3389/fnins.2024.1414145 (PMC11310129; doi:10.3389/fnins.2024.1414145)

Supplemental figure 1

a

Human (p.3866)	RAPQAQPTPGPGPA
Mus musculus (p.3882)	RAPQAQTTPGPGPA

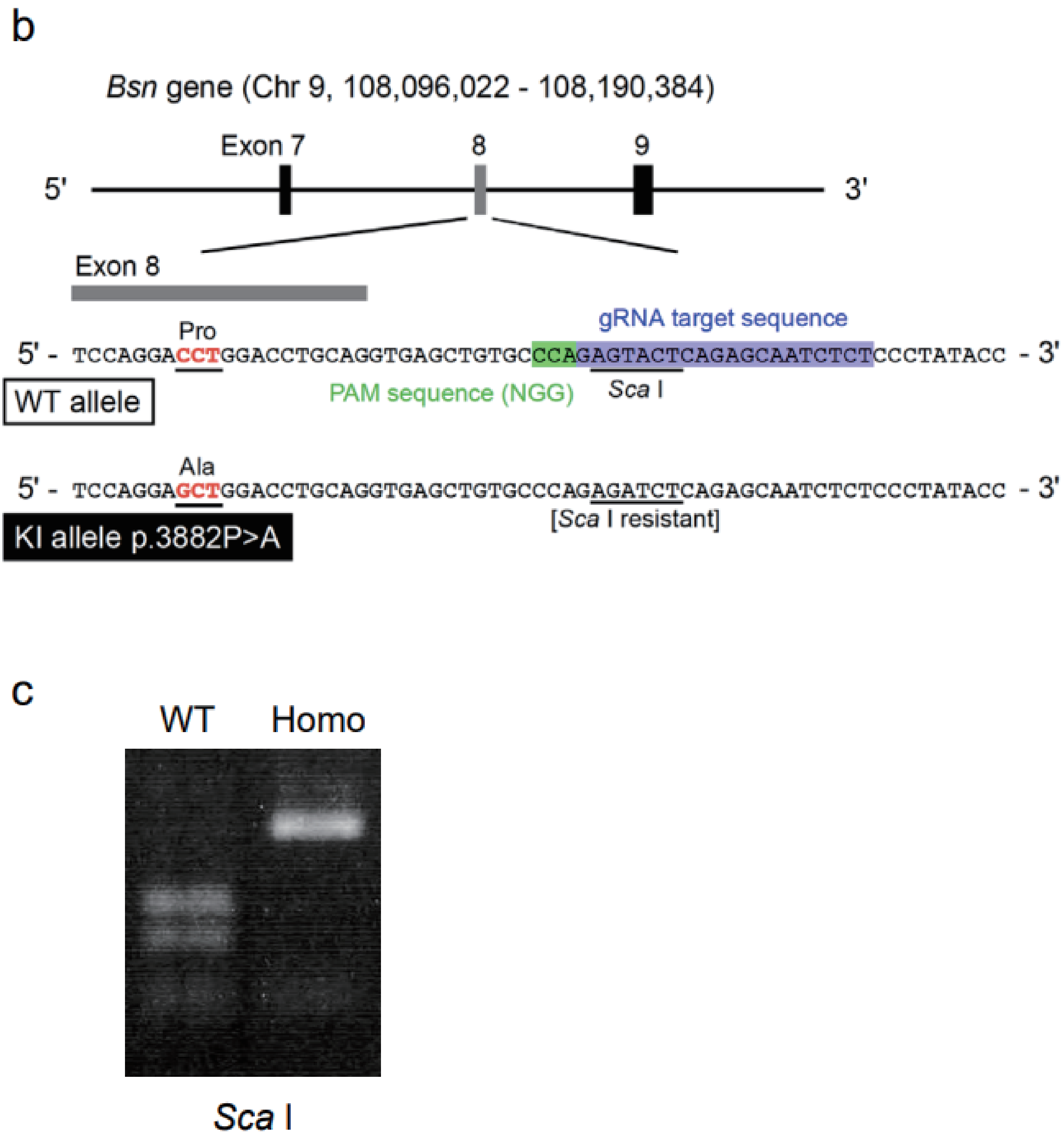

Supplemental figure 2

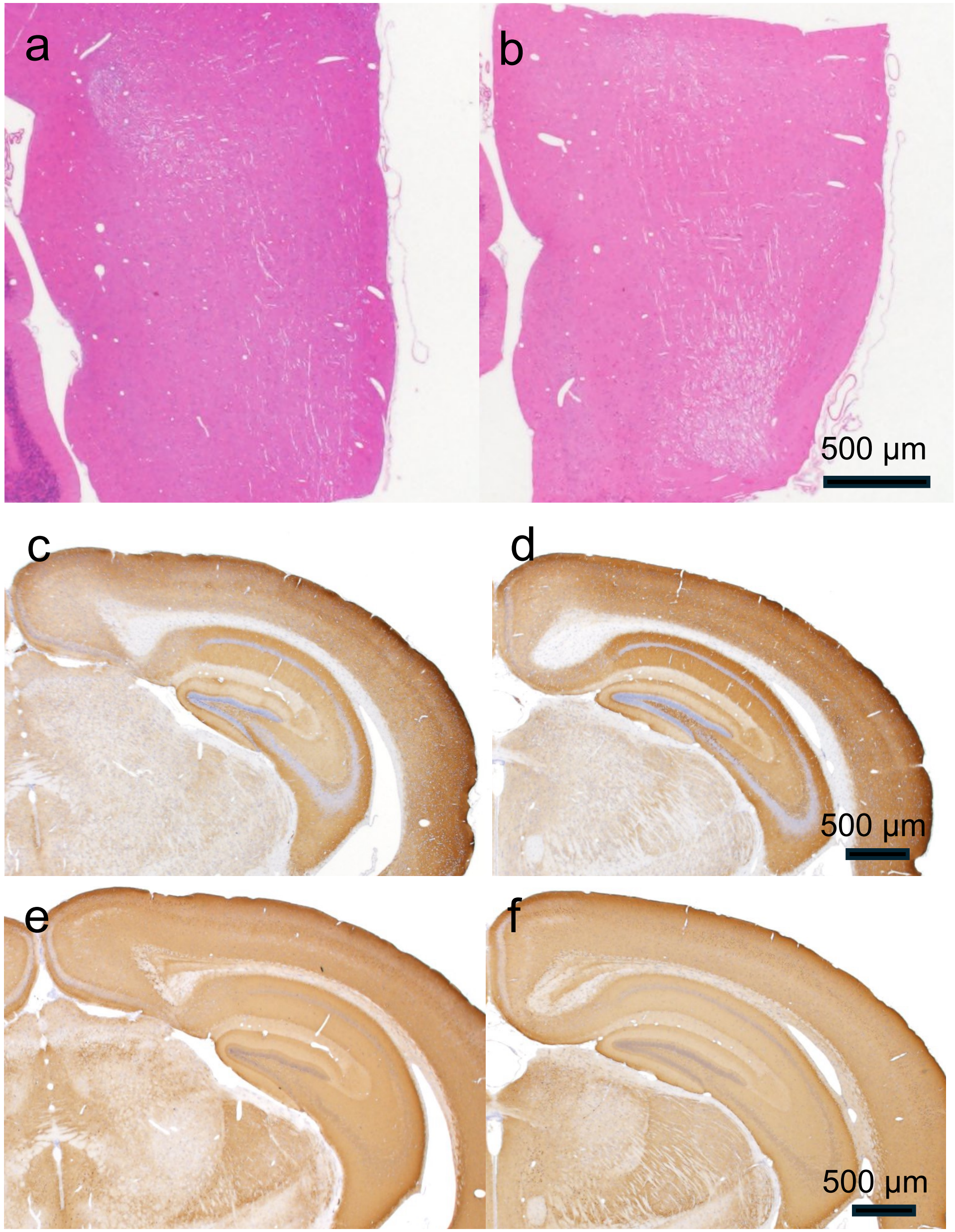

Supplemental figure 3

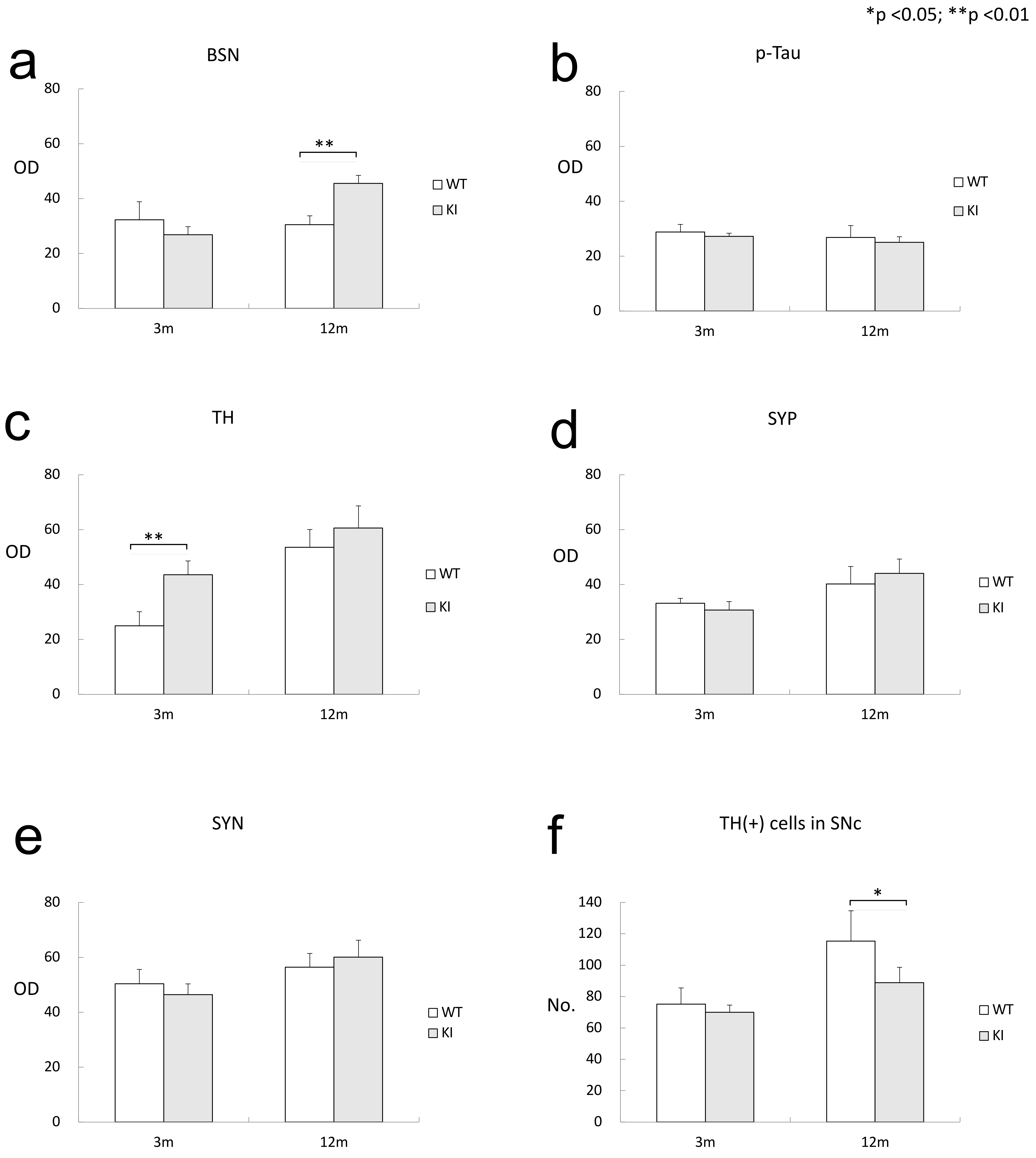

Supplemental figure 4

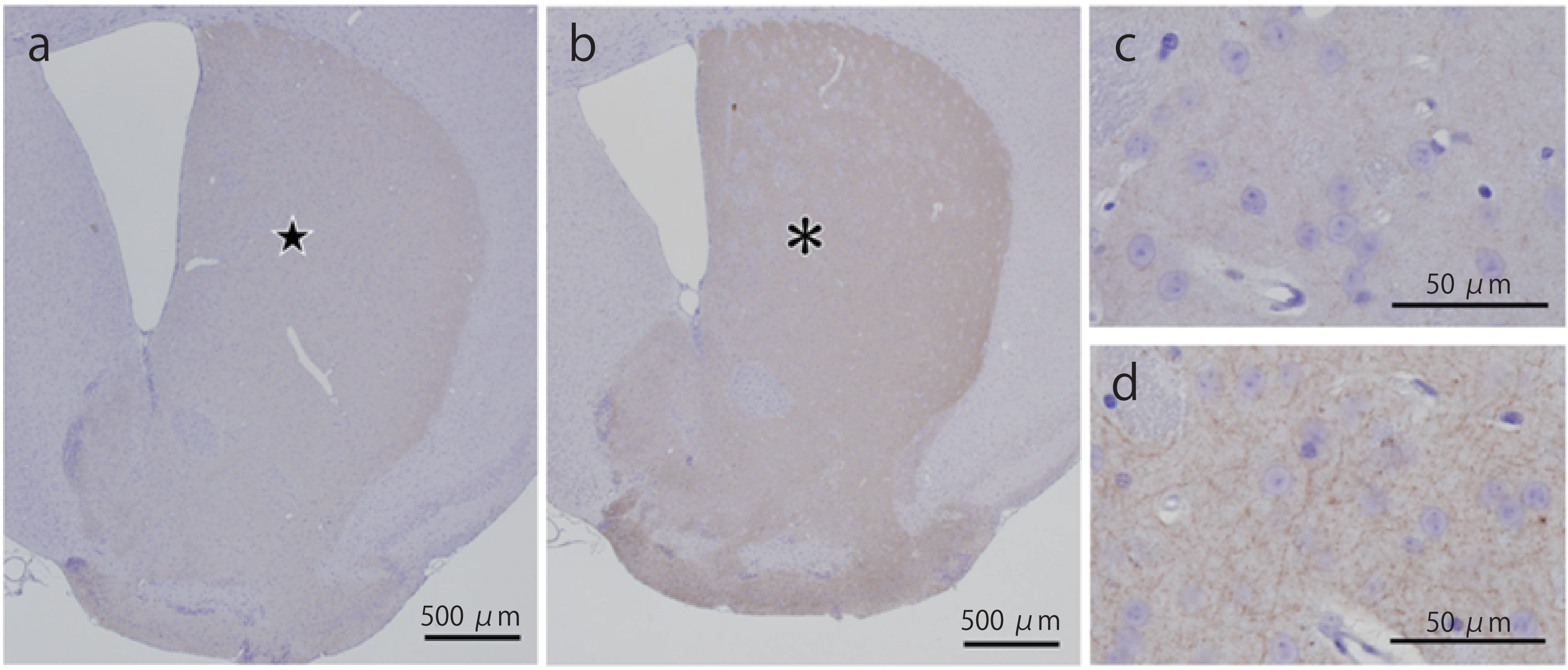

Supplemental figure 5

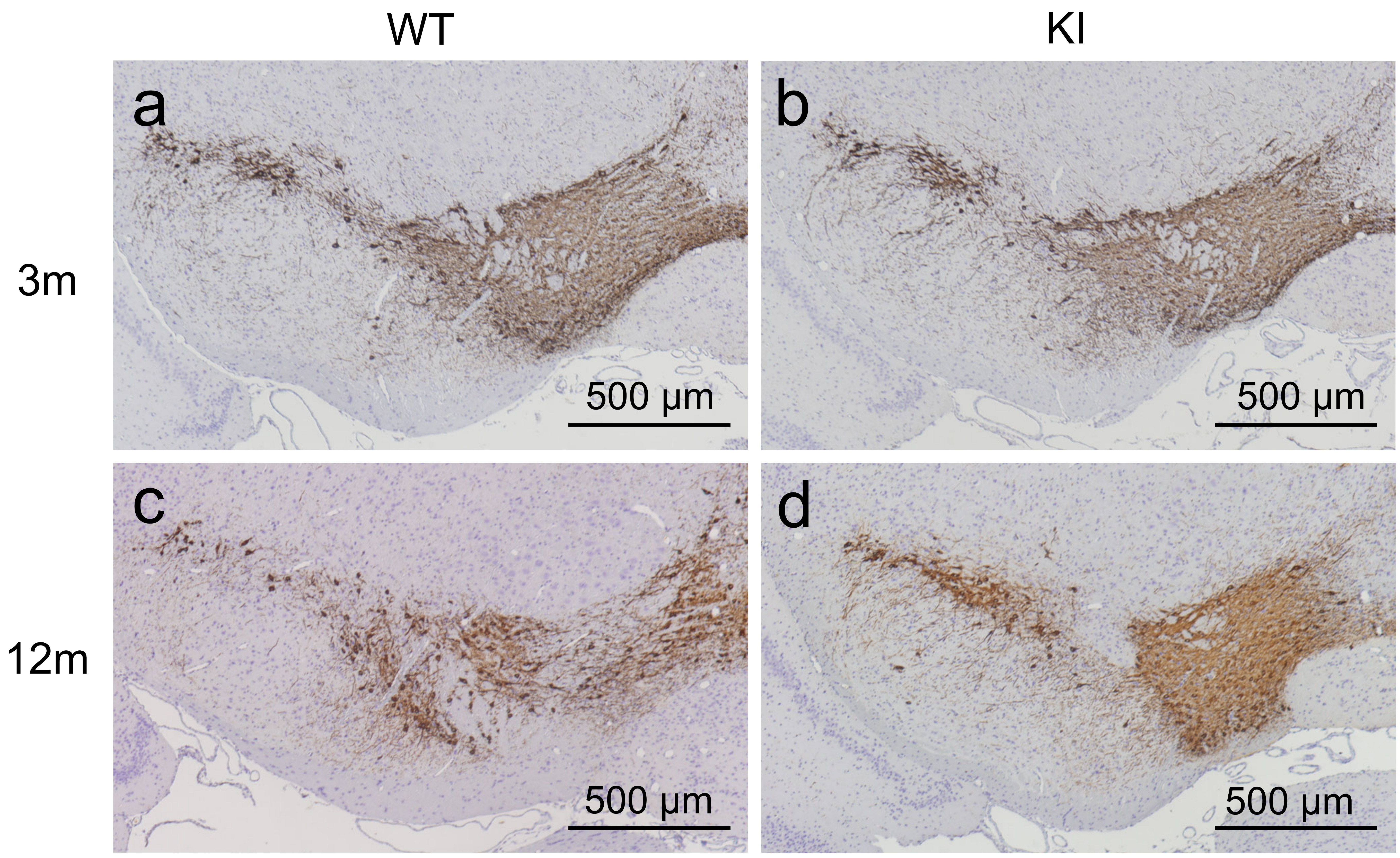

Supplemental figure 6

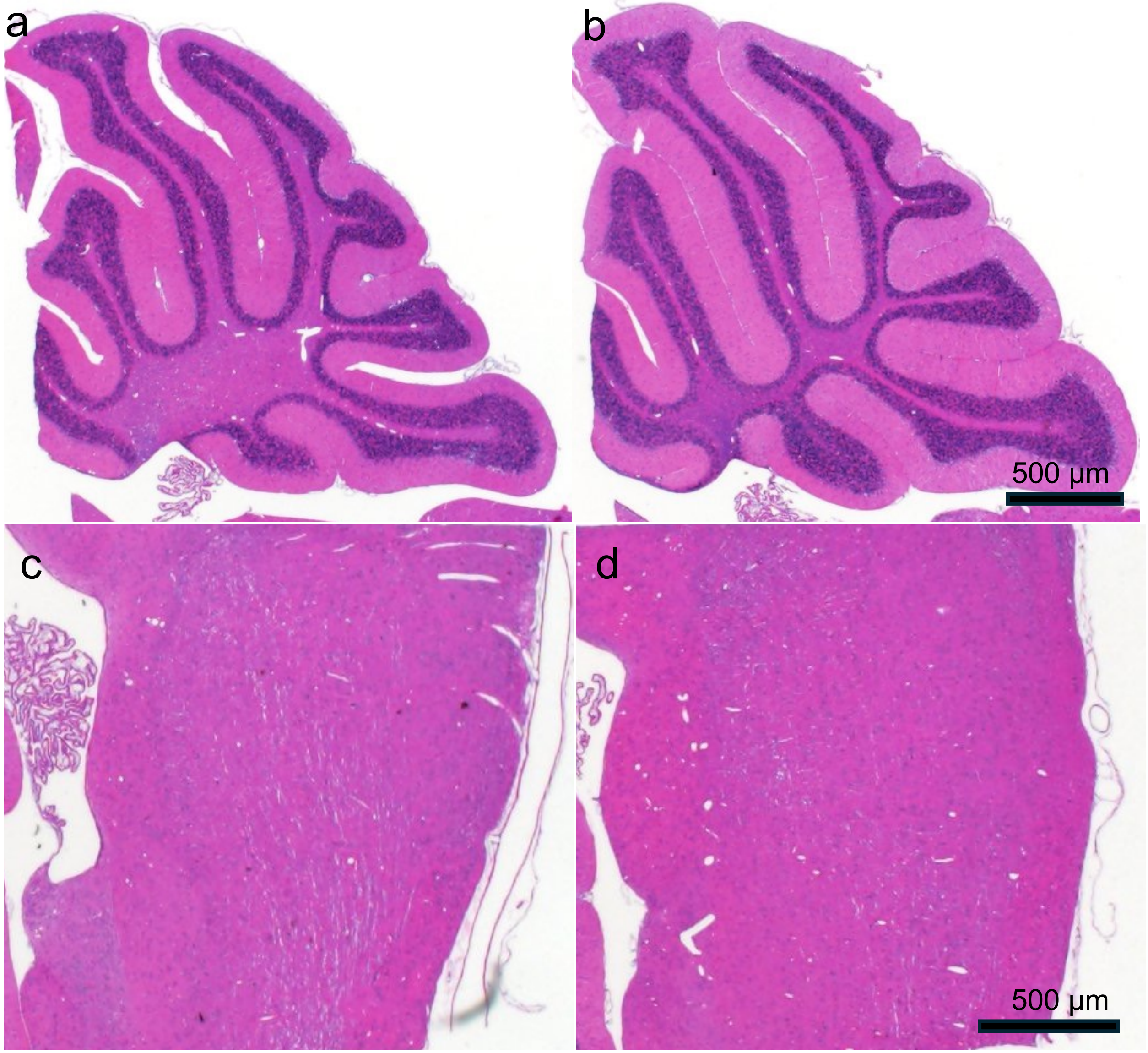

Supplemental figure 7

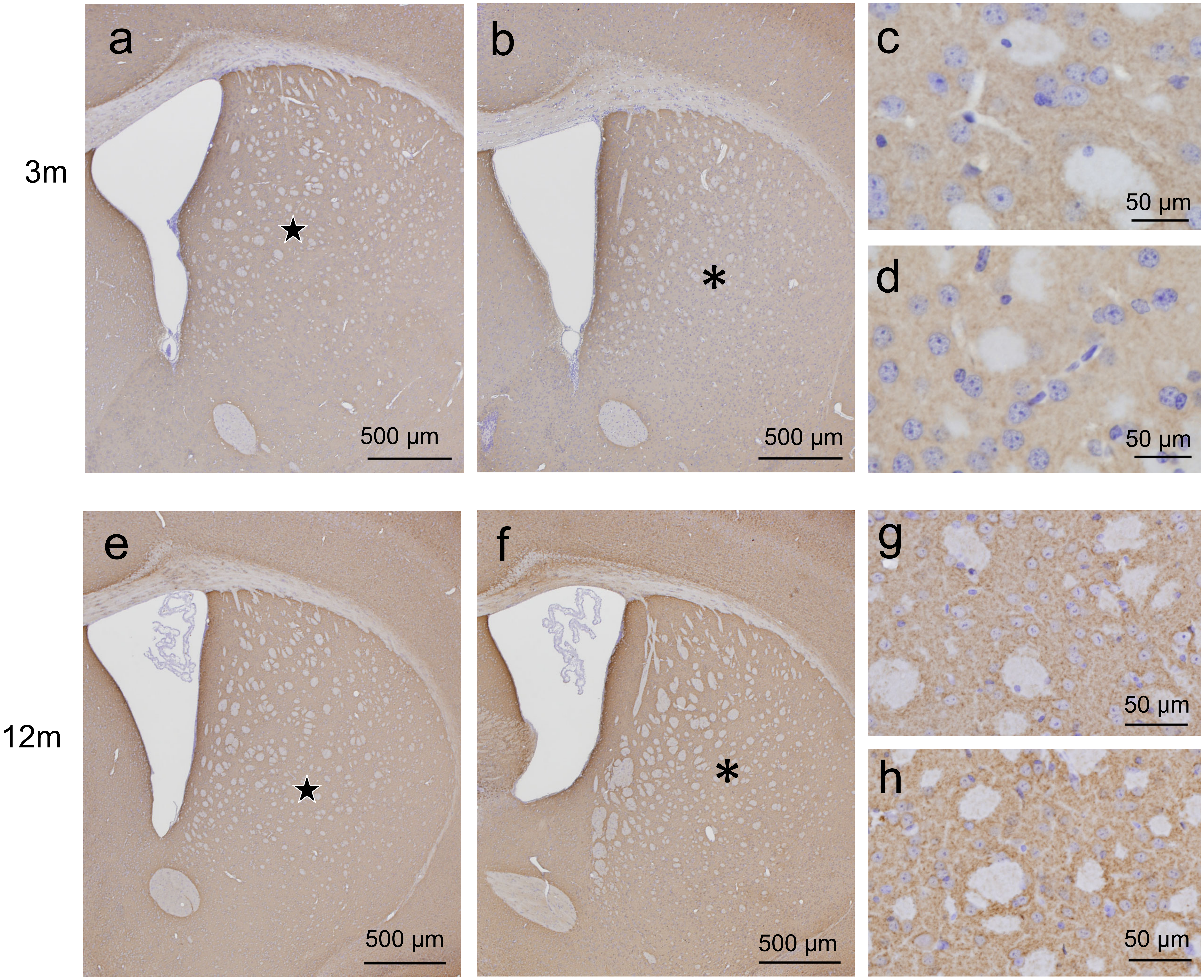

Supplemental figure 8

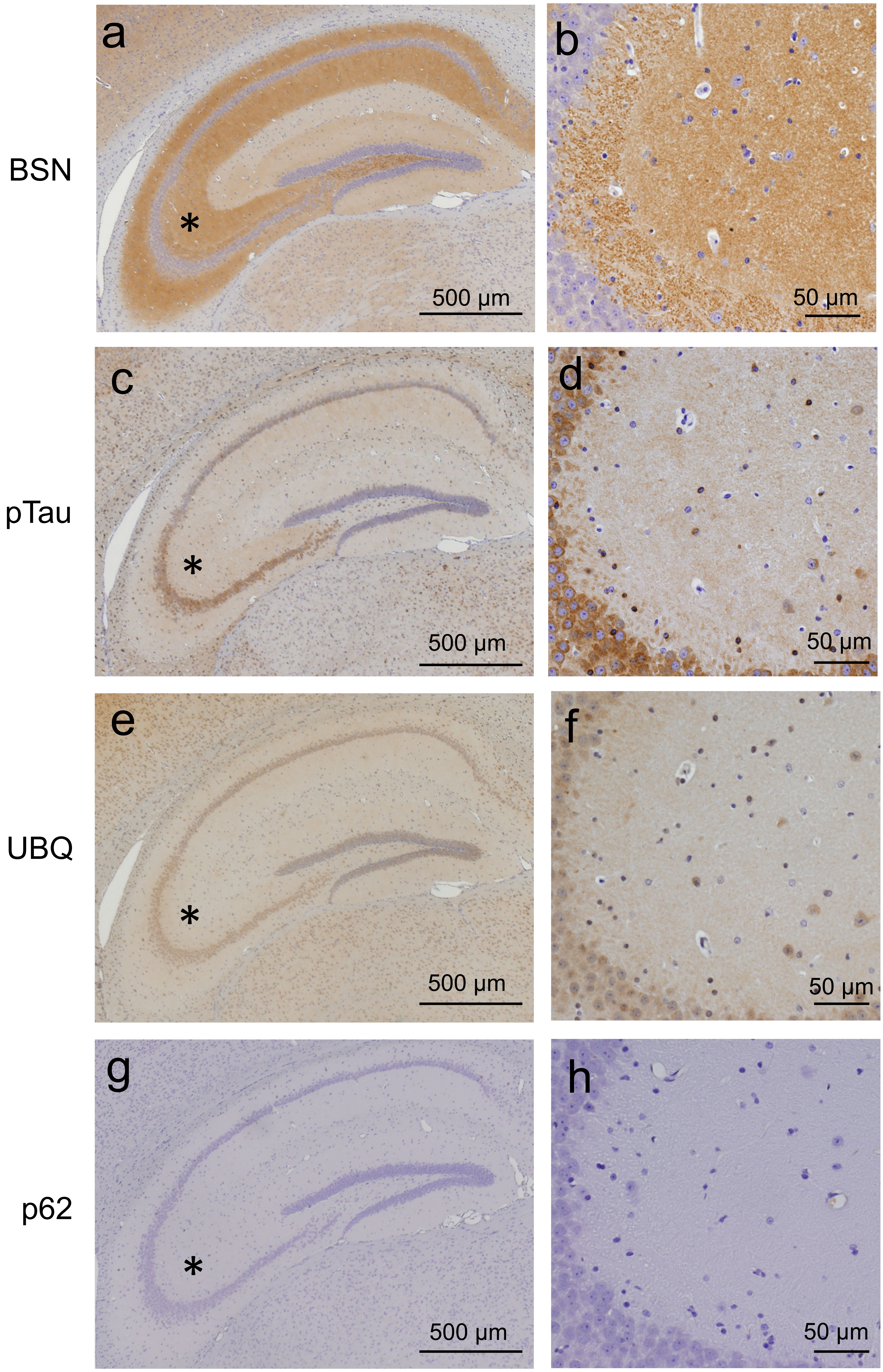

Supplemental figure 9

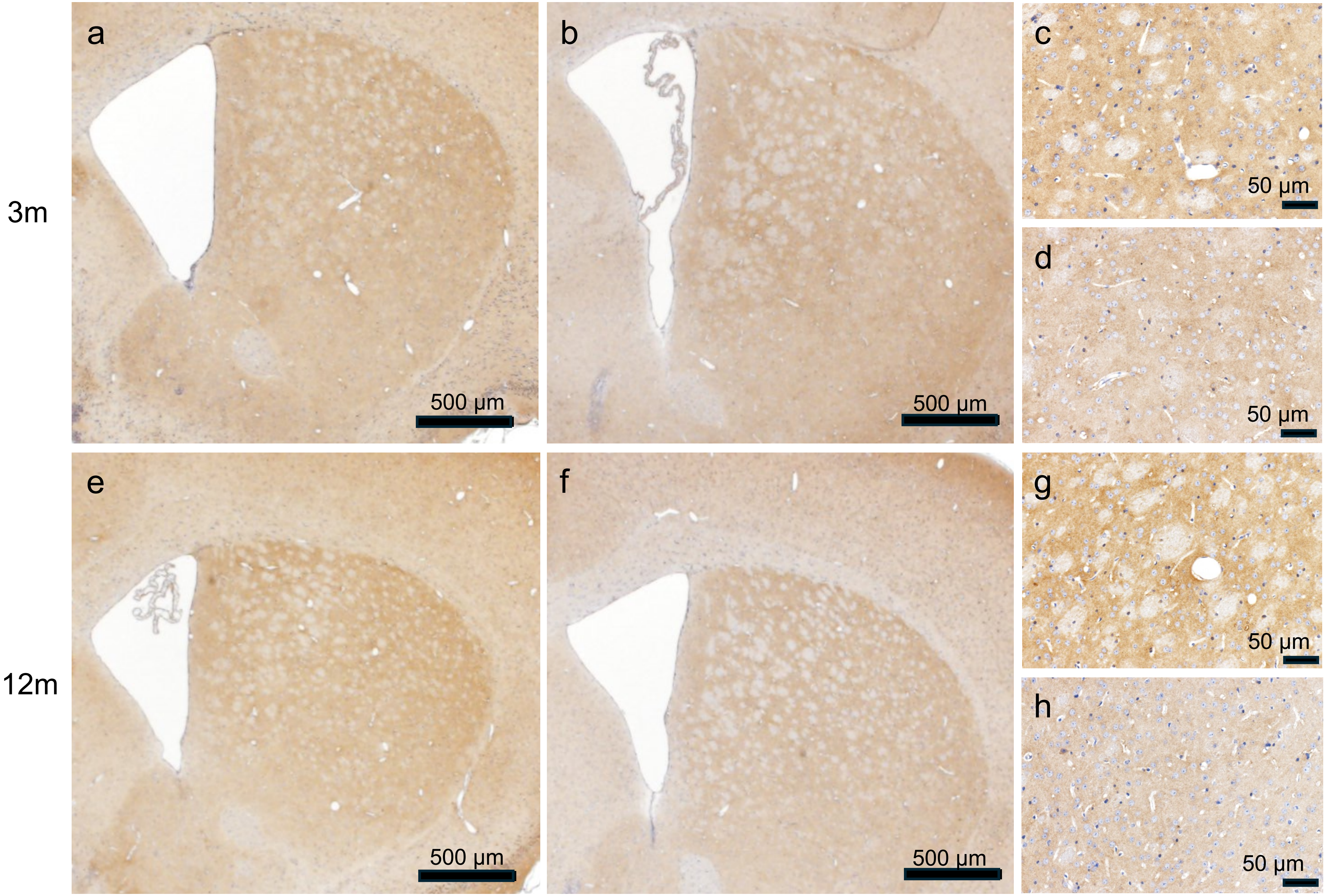

Supplement: Supplementary file 3 [file Image_1.pdf]
